# Diverse Role of TGF-β in Kidney Disease

**DOI:** 10.3389/fcell.2020.00123

**Published:** 2020-02-28

**Authors:** Yue-Yu Gu, Xu-Sheng Liu, Xiao-Ru Huang, Xue-Qing Yu, Hui-Yao Lan

**Affiliations:** ^1^Guangdong Provincial Key Laboratory of Clinical Research on Traditional Chinese Medicine Syndrome, Department of Nephrology, Guangdong Provincial Hospital of Chinese Medicine, The Second Affiliated Hospital, Guangzhou University of Chinese Medicine, Guangzhou, China; ^2^Department of Medicine and Therapeutics, Li Ka Shing Institute of Health Sciences, The Chinese University of Hong Kong, Hong Kong, China; ^3^Guangdong-Hong Kong Joint Laboratory for Immunity and Genetics of Chronic Kidney Disease, Guangdong Academy of Medical Sciences, Guangdong Provincial People‘s Hospital, Guangzhou, China

**Keywords:** TGF-β, Smads, fibrosis, inflammation, mechanisms, therapy

## Abstract

Inflammation and fibrosis are two pathological features of chronic kidney disease (CKD). Transforming growth factor-β (TGF-β) has been long considered as a key mediator of renal fibrosis. In addition, TGF-β also acts as a potent anti-inflammatory cytokine that negatively regulates renal inflammation. Thus, blockade of TGF-β inhibits renal fibrosis while promoting inflammation, revealing a diverse role for TGF-β in CKD. It is now well documented that TGF-β1 activates its downstream signaling molecules such as Smad3 and Smad3-dependent non-coding RNAs to transcriptionally and differentially regulate renal inflammation and fibrosis, which is negatively regulated by Smad7. Therefore, treatments by rebalancing Smad3/Smad7 signaling or by specifically targeting Smad3-dependent non-coding RNAs that regulate renal fibrosis or inflammation could be a better therapeutic approach. In this review, the paradoxical functions and underlying mechanisms by which TGF-β1 regulates in renal inflammation and fibrosis are discussed and novel therapeutic strategies for kidney disease by targeting downstream TGF-β/Smad signaling and transcriptomes are highlighted.

## Introduction

Increasing evidence shows that chronic kidney disease (CKD) is a global-burden-disease (Romagnani et al., 2017). The prevalence and incidence of CKD have risen by almost 90% over last 30 years (Provenzano et al., 2019). During the progression of CKD, renal function is impaired with a loss of nephrons and the development of renal fibrosis characterized by the excessive accumulation of extracellular matrix (ECM) components, reduction in glomerular filtration rate (GFR), and abnormal albuminuria (Glassock et al., 2017). CKD eventually leads to the development of end-stage renal disease (ESRD) (Eddy and Neilson, 2006; Liu, 2011). Fibrosis and inflammation are the two major features of CKD and prolonged renal inflammation promotes renal fibrosis as well (Meng et al., 2014; Li et al., 2017). Physiologically, fibrosis is a repair and healing process in response to the initial renal insults. However, as the pathological condition prolongs, unresolved renal inflammation turns into a major driving force to promote renal scar formation via a progressive process of renal fibrosis (Meng et al., 2014; Mihai et al., 2018).

**FIGURE 1:**
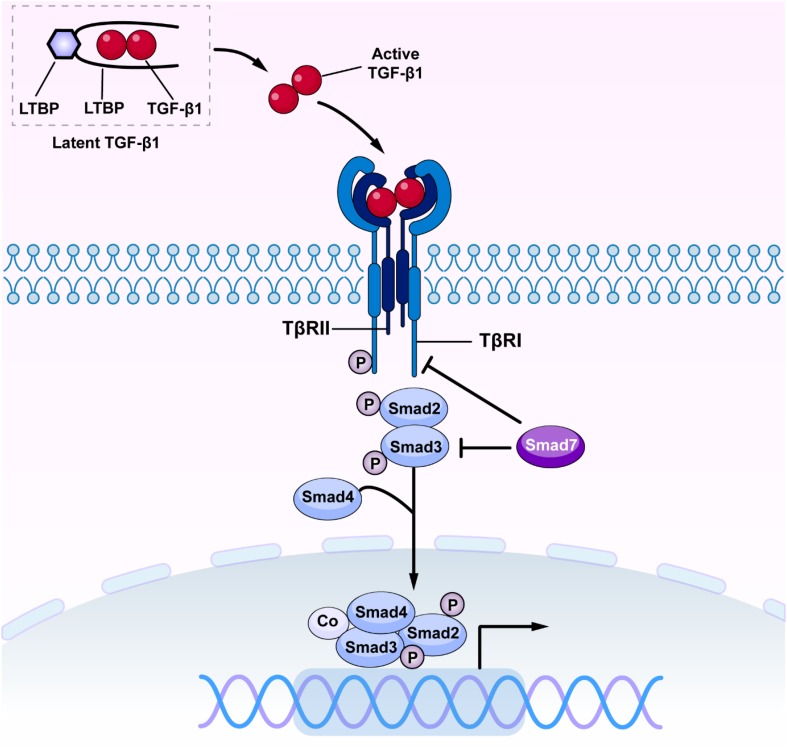
The canonical TGF-β/Smad signaling in fibrosis. Once released, active TGF-β1 binds TβRII and activates TβRI and R-Smads (Smad2 and Smad3), resulting in formation of a complex with Smad4. The Smad2/3/4 complex then translates into the nucleus and binds to the target genes to induce fibrosis and inflammation. TGF-β, transforming growth factor β; TβRI, TGF-β receptor type I; TβRII, TGF-β receptor type II.

Transforming growth factor-β has been long considered as a master cytokine in the pathogenesis of renal inflammation and fibrosis (Meng et al., 2016). The TGF-β superfamily contains members of TGF-βs, activins, inhibins, growth and differentiation factors (GDFs), bone morphogenetic proteins (BMPs), and glial-derived neurotrophic factors (GDNFs) (Zhang and Newfeld, 2013). It is well established that there are three isoforms of TGF-β in mammals, the TGF-β1, 2 and 3 (Roberts et al., 1991). Of these, TGF-β1 has been considered as a profibrotic mediator in various kidney diseases (Sureshbabu et al., 2016). Newly synthesized TGF-β1 releases and binds to the latency-associated peptide (LAP) to form a latent complex which later binds to the TGF-β binding protein (LTBP) to form a larger complex (Ando et al., 1995; Kusakabe et al., 2008). The latent complex is inactive and stored in the ECM until it is released by reactive oxygen species (ROS) and plasmin or acid. Once TGF-β1 is released from LAP and LTBP, it becomes active (Saharinen et al., 1999; Annes et al., 2003). Active TGF-β1 binds to Type II TGF-β receptor (TβRII), which recruits and activates Type I TGF-β receptor (TβRI) and downstream receptor-associated Smads (R-Smads), Smad2, and Smad3. The phosphorylated Smad2/3 then form an oligomeric complex with Smad4 (Derynck and Zhang, 2003; Lan and Chung, 2012). Subsequently, the Smad2/3/4 complex translocate into the nucleus to regulate transcription of target genes, inducing α-smooth muscle actin (α-SMA), collagens, and inhibitory Smad7 (Nakao et al., 1997; Miyazawa and Miyazono, 2017). Interestingly, Smad7 can antagonize TGF-β-mediated fibrosis, carcinogenesis and inflammation in various diseases (Yan et al., 2009; Troncone et al., 2018; Zhou G. et al., 2018). Smad7 negatively regulates TGF-β/Smad signaling by competing with the R-Smad binding to the TβRI (Yan et al., 2016; Figure 1). Moreover, Smad7 also induces the IκBα, a NF-κB inhibitor, to suppress NF-kB-driven inflammatory response (Bitzer et al., 2000; Wang et al., 2005a; Chen et al., 2018).

In this review, the diverse roles of canonical TGF-β signaling, the distinct roles of downstream Smad proteins, and the potential therapeutic strategies for renal fibrosis and inflammation by targeting downstream TGF-β/Smad signaling are discussed.

## Diverse Roles of TGF-β1 in Renal Fibrosis and Inflammation

It is well accepted that TGF-β is a master regulator in renal inflammation and fibrosis (Meng et al., 2016). TGF-β exerts multifunctional effects on cell proliferation, apoptosis, migration, differentiation, and ECM production (Massagué, 2012). TGF-β1 induces tubular and glomerular epithelial cell-to-mesenchymal transition (EMT) and excessive ECM production and deposition in glomeruli and tubulointerstitium (Fan et al., 1999; Ng et al., 1999). TGF-β1 is highly expressed in a wide range of kidney diseases associated with fibrosis (Lopez-Hernandez and Lopez-Novoa, 2012; Wang et al., 2017; Isaka, 2018). The functions of TGF-β1 on renal fibrosis and EMT were further confirmed by the findings that overexpression of active TGF-β1 in liver causes the development of severe renal fibrosis in mice (Bottinger et al., 1996; Kopp et al., 1996). Whereas, anti-TGF-β treatments by using neutralizing antibodies (Border et al., 1990), inhibitors against the TβRII (Sutaria et al., 1998; Liu et al., 2018), or antisense oligonucleotides to TGF-β1 (Akagi et al., 1996; Miyajima et al., 2000; Ziyadeh et al., 2000; Chen et al., 2003) halt the progression of renal fibrosis, suggesting a vital pathological role of TGF-β in CKD.

Renal inflammation is driven by NF-κB-dependent mechanism (Sanz et al., 2010; Ernandez and Mayadas, 2016). TGF-β is considered to be one of anti-inflammatory cytokines during the renal repair process in response to the injuries (Meng et al., 2014; Nikolic-Paterson et al., 2014; Meng, 2019; Tang et al., 2019). A number of studies have reported that mice deficient TGF-β1 suffer from the lethal inflammation and the early death (Kulkarni et al., 1993; Yaswen et al., 1996), suggesting a protective role for TGF-β in renal inflammation. Consistently, conditional deletion of TβRII from mice results in protection against TGF-β/Smad3-mediated renal fibrosis while enhancing NF-κB-driven renal inflammation (Meng et al., 2012a). More importantly, TGF-β is also a master regulator of T cell immune responses in a variety of immune diseases (Li and Flavell, 2008), which makes TGF-β as a key regulator in renal inflammation.

**FIGURE 2:**
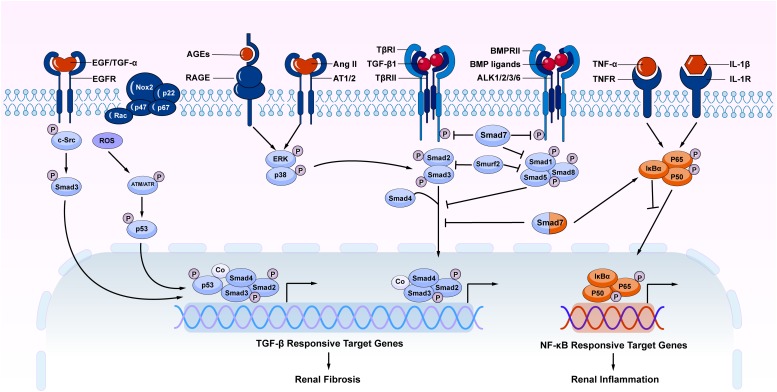
The overview of crosstalk pathways associated with renal fibrosis and inflammation. Many stress molecules such as TGF-β1, EGF, TGF-α, ROS, AGEs, and Ang II can activate individual pathways and interact with TGF-β/Smad signaling pathway to regulate renal fibrosis and inflammation. Among TGF-β super family, the BMP signaling negatively regulates TGF-β/Smad signaling. In TGF-β/Smad signaling, Smad7 inhibits the phosphorylation of TβRI and R-Smads via ubiquitin degradation mechanism. Meanwhile, Smad7 also alleviates renal inflammatory by inducing IκBα, therefore inhibiting NF-κB-driven inflammation. AGEs, advanced glycation end products; RAGE, receptor for AGE; Ang II, angiotensin II; AT1/2, Ang II receptor 1 and 2; NF-κB, nuclear factor κ-light-chain-enhancer of activated B cells; EGF, epidermal growth factor; EGFR, EGF receptor; c-Src, proto-oncogene tyrosine-protein kinase Src; ROS, reactive oxygen species; BMP, bone morphogenic protein; ALK, activin receptor-like kinases; TNF-α, tumor necrosis factor α; TNFR, TNF receptor; IL-1, Interleukin 1; IL-1R, IL-1 receptor; Nox, NADPH oxidase.

It should be pointed out that TGF-β signaling is not the sole pathway mediating the fibrotic process (Luo, 2017). Increasing evidence shows that TGF-β signaling can interact with other signaling pathways to mediate fibrosis. Among TGF-β signaling, both canonical and non-canonical TGF-β/Smad signaling pathways play a role in the renal fibrosis (Figure 2). Importantly, under disease conditions, Smad signaling can also be activated independently TGF-β1 by many stress molecules such as angiotensin II, and advanced glycation end products (AGE) via the ERK/p38/MAPK-Smad crosstalk pathway (Wang et al., 2005b, 2006; Yang et al., 2009; Meng et al., 2016). TGF-β/Smad can also interact with other signaling pathways such as Wnt/β-catenin, Jagged1/Notch, and Hedgehog to regulate epithelial dedifferentiation, myofibroblast transformation and proliferation (Edeling et al., 2016). In addition, TGF-β can induce renal fibrosis by transactivating epidermal growth factor receptor (EGFR) and p53 via proto-oncogene tyrosine-protein kinase Src (c-Src) and ROS-dependent mechanisms (Samarakoon et al., 2013; Harskamp et al., 2016). TGF-β1 also induces phosphorylation and acetylation of p53 and promote formation of p53/Smad3 complexes during renal fibrosis (Higgins et al., 2018; Rane et al., 2019). By contrast, BMP signaling via Smad1/5/8 complex is able to counter regulate TGF-β/Smad-mediated renal fibrosis (Weiskirchen et al., 2009; Meng et al., 2013; Munoz-Felix et al., 2015). Thus, TGF-β may exert its diverse role in renal inflammation and fibrosis by interacting with many other signaling pathways and molecules.

## Distinct Roles of Smad2 and Smad3 in Renal Fibrosis

In canonical TGF-β signaling, Smad2, and Smad3 are two key downstream mediators that are highly activated in the fibrotic kidney (Wang et al., 2006; Chung et al., 2010b; Zhou et al., 2010; Loeffler et al., 2018). Although Smad2 and Smad3 bind together, their functional roles are distinct. In the context of fibrosis, Smad3 is pathogenic while Smad2 is protective (Meng et al., 2010, 2016; Duan et al., 2014). Smad3 can induce matrix deposition by directly binding to the promoter region of collagen-producing genes and tissue inhibitor of matrix metalloproteinases (TIMP) while reducing the activity of MMP-1 to inhibit ECM degradation (Hall et al., 2003). By contrast, role of Smad2 in fibrosis is not fully elucidated due to a lack of Smad2 knockout (KO) mice which is embryonic lethal (Ju et al., 2006). However, a recent finding that conditional deletion of Smad2 from TECs accelerates renal fibrosis reveals a protective role of Smad2 in renal fibrosis (Meng et al., 2010). In addition, FSP1-specific Smad2 knockout in renal tubular, endothelial, and interstitial cells is also reported to reduce renal fibrosis and epithelial-to-mesenchymal transition in murine streptozotocin (STZ)-induced diabetic nephropathy (Loeffler et al., 2018).

## Diverse Role of Smad4 in Renal Fibrosis and Inflammation

Smad4 is a common Smad associated with nuclear translocation of Smad2/3 and Smad1/5/8 complexes in response to TGF-β and BMP signaling (Gomez-Puerto et al., 2019). Limited evidence has shown a direct role of Smad4 in renal fibrosis due to the lethality of Smad4 knockout mice. However, conditional deletion of Smad4 from TECs significantly reduces renal fibrosis in the obstructive kidney (Meng et al., 2012b). Mechanistically, deletion of Smad4 inhibits renal fibrosis by suppressing Smad3 promoter activity and blocking the binding of Smad3 to the collagen promoter without affecting its phosphorylation and nuclear translocation (Meng et al., 2012b). This finding is consistent with studies in Smad4 knockout mesangial cells and in the folic acid-induced rodent model (Tsuchida et al., 2003; Morishita et al., 2014). It is also reported that the formation of Smad3/Smad4/CDK9 complex drives renal fibrosis during ureteral obstruction (Qu et al., 2015). In contrast, conditional deletion of Smad4 promotes renal inflammation by impairing Smad7-mediated inhibition of NF-κB activation (Meng et al., 2012b). Thus, Smad4 may play a diverse role in renal fibrosis and inflammation and may not be a specific therapeutic target for CKD.

## Smad7 as an Inhibitory Protein of Renal Fibrosis and Inflammation

Smad7 is a vital negative regulator of both TGF-β/Smad and NF-κB signaling pathways (Lan, 2008, 2011; Yan and Chen, 2011; Meng et al., 2016). Indeed, although TGF-β1 induces Smad7 transcriptionally, Smad7 inhibits TGF-β signaling by directly binding to the TβRI and blocking the activation of R-Smads (Hayashi et al., 1997). Mechanistically, Smad7 interacts with E3 ubiquitin ligases, such as arkadia, Smurf1 or Smurf2 (Smad ubiquitination regulatory factors), and recruit them to the TRβI to cause its degradation, hence resulting in the inhibition of TGF-β/Smad signaling (Ebisawa et al., 2001; Chong et al., 2006; Liu et al., 2008). Under fibrosis conditions, Smad7 is reduced while Smad3 is highly activated as seen in diabetic nephropathy, hypertensive nephropathy, and aristolochic acid-induced nephropathy (Chen et al., 2011; Liu et al., 2012; Chung et al., 2013a; Tian et al., 2015). Thus, the imbalance between Smad3 and Smad7 signaling may be a key mechanism in fibrogenesis and rebalancing this pathway by overexpressing Smad7 and inactivating Smad3 may represent as a better therapeutic strategy for CKD.

Smad7 can also induce expression of IκBα, an inhibitor of NF-κB, to negatively regulate NF-κB-driven renal inflammation (Wang et al., 2005a, b; Lan, 2008, 2011). Furthermore, Smad7 can interact with NF-κB directly as Smad7 promoter contains a putative NF-κB regulatory site (Nagarajan et al., 2000). Under CKD conditions, loss of renal Smad7 is associated with activation of NF-κB signaling and severe renal inflammation as reported in hypertensive nephropathy (Liu et al., 2013, 2014) and aristolochic acid-induced nephropathy (Dai et al., 2015). In contrast, overexpression of Smad7 suppresses both renal fibrosis and inflammatory in these disease models, making Smad7 as an promising therapeutic strategy for CKD (Lan, 2008).

## Diverse Role of TGF-β/Smad Signaling in Regulation of Non-Coding RNAs Expression and Functions During Renal Fibrosis and Inflammation

MicroRNAs (miRNAs) are small (approximately 20–22 nucleotides in length) non-coding single stranded RNAs. More than 200 miRNAs have been identified in renal cells and tissues so far (Jelencsics and Oberbauer, 2015). These miRNAs regulate a wide range of biological processes, including fibrosis and inflammation. Increasing evidence has demonstrated that TGF-β1/Smad3 signaling regulates various miRNAs during the renal pathological processes (Meng et al., 2016; Tang et al., 2018). As a transcriptional factor, Smad3 can bind and upregulate or downregulate miRNAs to promote renal inflammation and fibrosis. It is now clear that Smad3, but not Smad2, regulates these miRNAs by physically interacting with Smad binding site (SBE) located in their promoters to either increase (such as miR-21 and miR-192) or inhibit their transcription (such as miR-29 and miR-200 families) (Chung and Lan, 2015). In addition, Smad7 may inactivate Smad3 to protect kidneys from fibrosis by upregulating renal miR-29b but suppressing miR-192 and miR-21 (Chung and Lan, 2015). Among these miRNAs, miR-21 is well characterized as a profibrotic miRNA. miR-21 is upregulated in renal fibrosis in the patients with CKD as well as AKI (Zarjou et al., 2011; Chau et al., 2012; Glowacki et al., 2013). Mice deficient miR-21 or administration of anti-miR-21 oligonucleotides are able to protect against renal fibrosis (Zhong et al., 2011, 2013). Expression of miR-21 is positively regulated by Smad3 but negatively by Smad7 (Chung et al., 2013a). Overexpression of miRNA-21 promotes renal fibrosis by targeting PTEN and Smad7 (Zhou et al., 2013; McClelland et al., 2015). Thus, knockdown of miR-21 restores renal Smad7 levels and blocks both TGF-β/Smad3 and NF-κB signaling, thereby inhibiting progressive renal fibrosis and inflammation in mouse models of obstructive and diabetic nephropathy (Zhong et al., 2013). However, miR-21 may be also protective in kidney disease as miR-21-deficient TGF-β(1)-transgenic mice show increased proteinuria and glomerular injury in streptozotocin-induced diabetic mice, suggesting a diverse role of miR-21 as a feedback inhibitor of TGF-β/Smad3 signaling (Lai et al., 2015).

MiR-29 family is another well-documented miRNA in fibrotic diseases (He et al., 2013). The miR-29 family consists of miR-29a, b, c. All family members are encoded by two distinct genomic loci in both human and rodent genomes. As all members have the same seed binding sequence, they all bind to the same set of target genes (Kriegel et al., 2012). Renal miR-29b is decreased in association with activation of TGF-β/Smad3 signaling and progressive renal fibrosis in kidney diseases (Qin et al., 2011; Wang et al., 2012; Chen et al., 2014; Meng et al., 2016). miR-29b is negatively regulated by Smad3, but not Smad2, in response to TGF-β1, AGE, and angiotensin II (Qin et al., 2011; Wang et al., 2012; Chen et al., 2014; Yu et al., 2014; Zhang et al., 2014). Overexpression of miR-29 inhibits renal fibrosis and inflammation by targeting TGF-β and Sp1/NF-κB signaling (Chen et al., 2014; Zhang et al., 2014). Interestingly, miR-29b can also target T-bet, a master transcriptional factor for Th-1 T cell immune response. Therefore, overexpression of miR-29b is also capable of inhibiting T cell-mediated type-2 diabetic nephropathy in db/db mice (Chen et al., 2014). Notably, miR-29 also acts as a urinary exosome biomarker of renal fibrosis (Lv et al., 2013). Intramuscular injection of exosome-encapsulated miR-29 has been shown to inhibit renal fibrosis and muscle atrophy (Wang et al., 2019).

**FIGURE 3:**
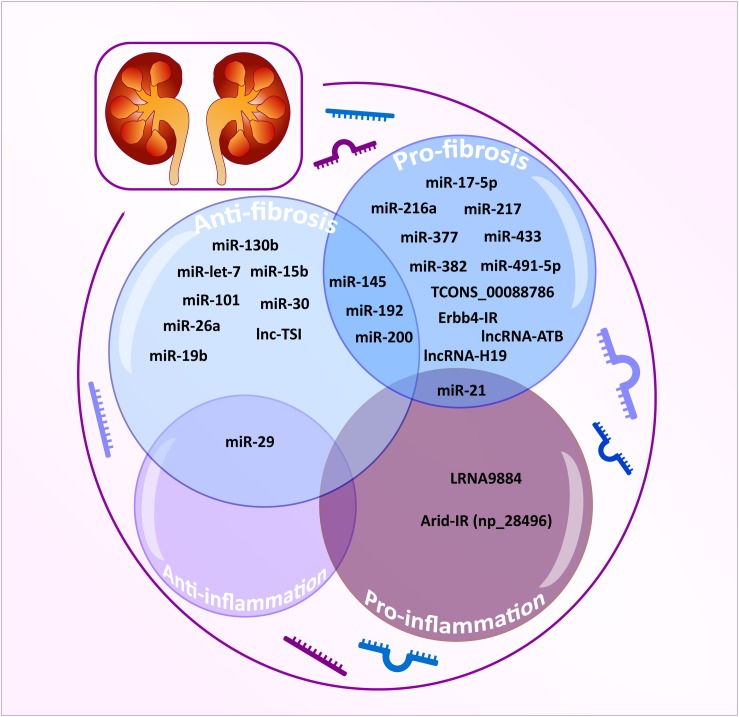
TGF-β/Smad3-dependent miRNAs and lncRNAs related to renal fibrosis and inflammation. TGF-β/Smad3-dependent miRNAs and lncRNAs are classified as anti-fibrotic (powder blue), pro-fibrotic (sky blue), anti-inflammatory (lavender), and pro-inflammatory effect (plum). The integrated area indicates multiple functions for each miRNA/lncRNA.

Moreover, miR-93, miR-216a, miR-217, miR-377, miR-382, miR-491-5p, miR-433 and miR-17-5p are also demonstrated to be TGF-β1/Smad3-regulated profibrotic miRNAs (Chung and Lan, 2015), whereas miR-let-7, miR-15b, miR-101, and miR-130b exert their antifibrotic effects by inhibiting the expression and activity of TβRI, thus limiting transduction of downstream TGF-β-mediated signals (Wang et al., 2014; Tang et al., 2018). Other miRNAs such as miR-19b, miR-26a, miR-29, and miR-30 inhibit the TGF-β1/Smad signaling by targeting Smads or fibrotic transcriptional factors (Tang et al., 2018). All these findings imply that TGF-β may regulate miRNAs to exert its diverse roles in renal inflammation and fibrosis as shown in Table 1 and Figure 3.

**TABLE 1:** MicroRNAs regulated by TGF-β/Smad signaling in renal fibrosis.

**Micro RNA**	**Target genes/Mechanisms**
**Antifibrotic**	
miR-15b	TβR1
miR-19b	TβR2
miR-26a	Smad4
miR-29	TGF-β1/2, Col, MMP, Fos, Adams, HDAC4
miR-30	TGF-β2, Snail
miR-101	TβR1
miR-130b	TβR1
miR-let-7	TβR1
**Antifibrotic or profibrotic**	
miR-145	TβR2, latent TGF-β1, KLF4
miR-192	P53, Zeb1/2E-cadherin
miR-200	TGF-β2, Zeb1/2E-cadherin
**Profibrotic**	
miR-17-5p	Smad7
miR-216a	PTEN
miR-217	PTEN
miR-377	SIRT1
miR-382	HSPD1, SOD2
miR-491-5p	Par-3
**Profibrotic and pro-inflammatory**	
miR-21	Smad7, PPARα, PTEN, ERK/MAPK, Spry1

However, the off-target effects, non-specificity, and toxicity of miRNAs are unavoidable. Thus, research into long non-coding RNAs (lncRNAs) is more promising for a better understanding of the pathogenic mechanisms of kidney diseases (Moghaddas Sani et al., 2018). Compared to miRNAs, lncRNAs are transcripts with lengths exceeding 200 nucleotides without protein-coding functions and are highly tissue-and-cell-type-specific. lncRNA regulates both target DNAs/RNAs and proteins transcriptionally or post-transcriptionally (Dykes and Emanueli, 2017). By using the high-throughput RNA sequencing, 21 TGF-β/Smad3-dependent lncRNAs have been identified in an immunologically induced anti-glomerular basement membranous glomerulonephritis (anti-GBM GN) and obstructive nephropathy (Zhou et al., 2014). Of these, the Arid-IR is a novel and Smad3-related lncRNA as a Smad3 binding site is found in its promoter region. It has been proven that knockdown of Arid2-IR in TECs improves renal inflammation *in vivo* and *in vitro* by inhibiting NF-κB-dependent inflammatory transduction without affecting Smad3-mediated fibrosis (Zhou et al., 2015). In contrast, Erbb4-IR is another novel Smad3-dependent lncRNA capable of inhibiting renal fibrosis by targeting miR-29b and Smad7 in both obstructive nephropathy and type II diabetic nephropathy, respectively (Feng et al., 2018; Sun et al., 2018). A recent study also reveals the pathogenic role and mechanism of LRNA9884 in type II diabetic nephropathy (Zhang et al., 2019). LRNA9884 is tightly regulated by Smad3 in response to TGF-β and AGEs and functions to trigger MCP-1 production by directly binding to the MCP-1 promoter, thereby promoting inflammation-driven type II diabetic nephropathy (Zhang et al., 2019). In addition, several TGF-β/Smad3-associated lncRNAs are found to be associated with renal fibrosis. TCONS_00088786 and TCONS_01496394 are TGF-β/Smad3-associated lncRNAs as they contain potential binding sites for Smad3 and silencing TCONS_00088786 inhibits renal interstitial fibrosis by targeting miR-132 (Sun et al., 2017; Zhou S.G. et al., 2018). lncRNA-ATB is highly upregulated in patients with acute renal allograft rejection and renal carcinoma and is able to promote EMT (Qi et al., 2017; Qiu et al., 2017; Zhou and Jiang, 2019). lncRNA uc.412 is able to induce mesangial cell proliferation *in vitro* although the underlying mechanisms are unclear (Yu et al., 2019). Lnc RNA-H19 is associated with TGF-β2-induced fibrosis *in vivo* and *in vitro* (Xie et al., 2016). lncRNA ENST00000453774.1 (LncRNA 74.1) is significantly down-regulated in TGF-β-treated TECs and in fibrotic kidney (Xiao et al., 2019). Interestingly, a recent study also revealed that decreased human lnc-TSI (TGF-β/Smad3-interacting long non-coding RNA) correlates with the degree of renal fibrosis in patients with IgA nephropathy and treatment with lnc-TSI inhibits renal fibrosis by blocking its binding to the MH2 domain of Smad3 (Wang et al., 2018).

Taken together, TGF-β may diversely regulate renal fibrosis and inflammation via Smad3-dependent miRNAs/lncRNAs as shown in Table 2 and Figure 3.

**TABLE 2:** Long non-coding RNAs regulated by TGF-β/Smad signaling in renal fibrosis.

**Non-coding RNA**	**Target genes/Mechanisms**
**Antifibrotic**	
Lnc-TSI	Smad3
**Antifibrotic or profibrotic**	
TCONS_01496394	Unclear
**Profibrotic**	
Erbb4-IR (np_5318)	miR-29b, Smad7
lncRNA-H19	miR-17
lncRNA-ATB	Livin
TCONS_00088786	miR-132
**Pro-inflammatory**	
LRNA9884	MCP-1
Arid2-IR (np_28496)	NF-κB

## Clinical Trials of Anti-TGF-β Therapy

Theoretically, TGF-β is a key mediator for renal fibrosis and thus targeting TGF-β signaling could be a good therapeutic strategy for CKD. There are many approaches to develop anti-TGF-β treatment for CKD clinically (Table 3). It has been shown that treatment with Pirfenidone, a non-specific antifibrotic effect of TGF-β, can improve eGFR in the trials of DN and focal segmental glomerulosclerosis (FSGS) (Lancaster et al., 2017). Disappointingly, a recent clinical trial study using a humanized monoclonal neutralizing antibody against TGF-β1 (LY2382770) for treatment of patients with diabetic nephropathy has been proven no efficacy on the improvements of serum creatinine, estimated GFR (eGFR), and proteinuria (Voelker et al., 2017). In addition, the use of another humanized monoclonal antibody, Fresolimumab that inhibits all three isoforms of TGF-β, also fails to achieve the endpoints of proteinuria reduction in patients with FSGS (Trachtman et al., 2011; Vincenti et al., 2017), demonstrating targeting on the upstream of TGF-β signaling may not be a good therapeutic strategy for CKD. It is possible that blockade of the general effect of TGF-β1, including latent form of TGF-β1, may attribute to the failure of these clinical trials. Our previous studies in latent TGF-β transgenic mice explain this notion since mice overexpressing latent TGF-β1 are protected against renal inflammatory and fibrosis in unilateral ureteral obstructive (UUO) nephropathy and anti-GBM glomerulonephritis model (Huang et al., 2008a, b). Thus, the latent form of TGF-β1 is renal protective while its active form is pathogenic. As most circulating TGF-β1 is latent form, thus, the use of anti-TGF-β1 antibodies may largely block the protective effect of latent TGF-β1, resulting in progressive renal injury as seen in these clinical trials. Results from these studies also suggest that treatment against renal fibrosis in patients with CKD should specifically target the downstream TGF-β signaling molecules, rather than to block the general effect of TGF-β1.

**TABLE 3:** Therapeutic drugs and clinical trials for treatment of CKD by targeting TGF-β.

**Drug and trials**	**Mechanisms**	**Disease**	**Drug administration and period**	**Results**	**Side effects**	**References**
**LY2382770**						
NCT01113801	TGF-β1	DN	Subcutaneous injection given monthly for 12 months	No efficacy on improvements in eGFR, Scr and proteinuria	Risk of toxicity and loss of renal efficacy	Voelker et al., 2017
**Fresolimumab**						
NCT01665391	TGF-β1,2,3	FSGS	Administered intravenously at 1 mg/kg or 4 mg/kg for 112 days, followed double-blind for 252 days	No efficacy in proteinuria reduction; non-significant trend on eGFR decline	Herpes zoster; skin lesions, bleeding events and cancers	Vincenti et al., 2017
NCT00464321	TGF-β1,2,3	FSGS	Administered intravenously at one of four single-dose (0.3,1,2 and 4 mg/kg), followed for 112 days	Less eGFR decline (non-significant)	Pustular rash	Trachtman et al., 2011
**Pirfenidone**						
NCT02689778	TGF-β1,2,3	DN	Administrated orally 600 mg with breakfast and 1200 mg with dinner for 12 months	Phase 3 ongoing	N/A	
NCT00063583	TGF-β1,2,3	DN	Administered orally at a dose of 1200 mg or 2400 mg per day for 12 months	eGFRs increased significantly in the 1200 mg/d pirfenidone group compared with placebo	Gastrointestinal disorders, fatigue and photosensitivity rash	Sharma et al., 2011
NCT02408744	TGF-β1,2,3	CKD	Prolonged-released tablets, orally administered 2 time per day for 36 months	Phase 2 ongoing	N/A	
NCT02530359	TGF-β1,2,3	Septic AKI	Pirfenidone extended release 600 mg per month every 12 h for 7 days	Phase 4 ongoing	N/A	
NCT00001959	TGF-β1,2,3	FSGS	Orally administrated 3 times daily for 12 months	Improved eGFR decline; no effect on BP or proteinuria	Dyspepsia, sedation, and photosensitive dermatitis	Cho et al., 2007

*DN, diabetic nephropathy; FSGS, focal and segmental glomerulosclerosis; CKD, chronic kidney disease; AKI, acute kidney disease.*

## Treatment of CKD by Targeting Downstream TGF-β/Smad Signaling Molecules and Non-Coding RNAs

**FIGURE 4:**
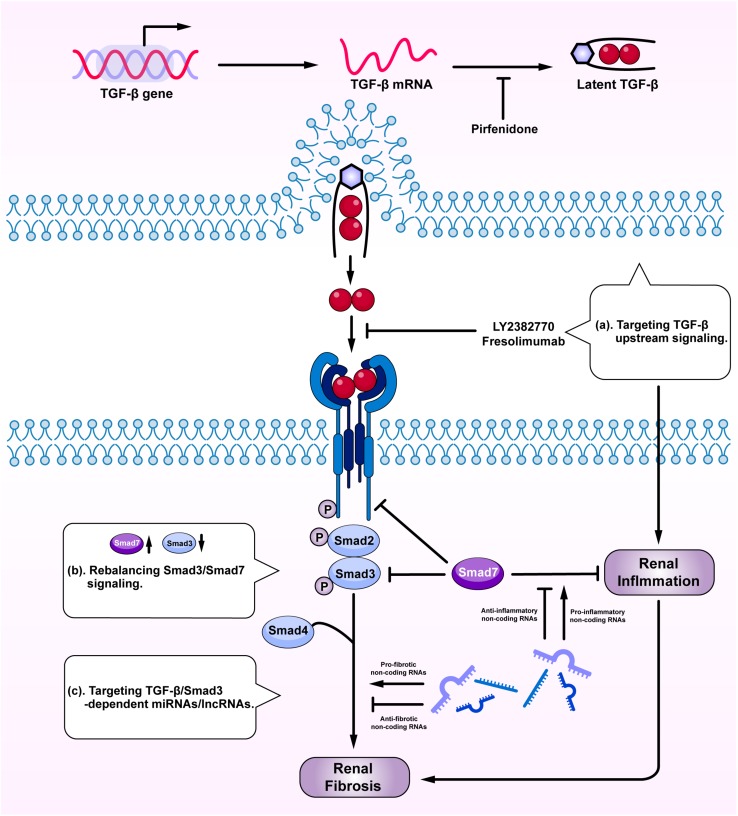
Therapeutic potentials by targeting TGF-β signaling. Anti-TGF-β treatment by: (a) targeting upstream signaling; (b) rebalancing Smad3/Smad7 signaling; and (c) targeting Smad3-dependent miRNAs/lncRNAs.

Given the diversity and the complexity of TGF-β in renal fibrosis and inflammation, direct targeting TGF-β or receptors may not be an ideal tactic due to its involvement in various vital biological processes (Trachtman et al., 2011; Vincenti et al., 2017; Voelker et al., 2017). Although general blockade of the upstream TGF-β signaling may reduce fibrosis, it can also promote renal inflammation and cause unexpected renal injuries (Figure 4a). Because the imbalance of TGF-β/Smad3 signaling with overreactive Smad3 and reduced Smad7 is a key mechanism leading to renal fibrosis and inflammation, rebalancing Smad3/Smad7 signaling may serve as effective strategies to treat renal fibrosis and inflammation (Figure 4b). SIS3, a specific Smad3-inhibitor, has been shown to inhibit renal fibrosis in STZ-induced diabetic nephropathy (Li et al., 2010) and in obstructive nephropathy (Zhang et al., 2018). Overexpression of renal Smad7 is also capable of inhibiting Smad3-mediated renal fibrosis and NF-κB-driven renal inflammation in various kidney diseases, including diabetic and hypertensive nephropathy (Chen et al., 2011; Lan, 2011; Ka et al., 2012; Liu et al., 2014), obstructive nephropathy (Li et al., 2002; Lan et al., 2003; Lan, 2008; Chung et al., 2013a), remnant kidney disease (Hou et al., 2005; Ng et al., 2005), crescentic glomerulonephritis (Ka et al., 2007), and chronic aristolochic acid nephropathy (Dai et al., 2015). Interestingly, treatment of CKD with two Traditional Chinese Medicine compounds, Naringenin from fruits as a Smad3 inhibitor and Asiatic acid derived from *Centella asiatica* as a Smad7 agonist, is capable of restoring the balance of Smad3/Smad7 signaling and thus additively inhibits renal fibrosis in rodent obstructive nephropathy (Meng et al., 2015). Similarly, the combination of Ginsenoside Rg1 from *Panax ginseng C. A. Mey* and Astragaloside IV from *Radix astragali* have also improved fibrosis and inflammation in STZ-induced diabetic nephropathy by inhibiting TGF-β/Smad2/3 while enhancing Smad7 signaling (Du et al., 2018). Asperulosidic acid, a bioactive iridoid glycoside, can also exert renal protective effects by inactivating both TGF-β/Smad and NF-κB signaling pathways (Xianyuan et al., 2019). Similar therapeutic effects are also found in other studies with herbal medicines (Nie et al., 2014; Wan et al., 2014; Zhao et al., 2016).

Targeting Smad3-dependent non-coding RNAs could be another therapeutic approach to treat renal fibrosis and inflammation (Figure 4c). Of Smad3-dependent miRNAs (Figure 3), inhibition of miR-21, miR-192, miR-433, and overexpression of miR-29 and miR-200 have been shown to have therapeutic effects on obstructive nephropathy (Chung et al., 2010a, 2013b; Oba et al., 2010; Qin et al., 2011; Zhong et al., 2011; Li et al., 2013) and diabetic nephropathy (Zhong et al., 2013; Chen et al., 2014). However, the off-target effect of anti-miRNA therapies raises concern and new therapeutic approach by targeting Smad3-dependent lncRNAs is sought. Targeting Arid2-IR and LRNA9884 can specifically inhibit renal inflammation while targeting Erbb4-IR can specifically inhibit renal fibrosis in obstructive and diabetic nephropathy (Zhou et al., 2015; Feng et al., 2018; Sun et al., 2018; Zhang et al., 2019). Furthermore, delivery of a human lncRNA lnc-TSI into the UUO kidney also inhibits Smad3-mediated renal fibrosis (Wang et al., 2018). All these findings highlight the therapeutic potentials by targeting downstream TGF-β signaling molecules including Smad3, Smad7, and non-coding RNAs in renal fibrosis and inflammation.

## Conclusion

Transforming growth factor-β plays diverse roles in renal fibrosis and inflammation. Blockade of upstream TGF-β signaling may not be a good therapeutic strategy, which has been proved by unsatisfied clinical trials. TGF-β may specifically regulate renal fibrosis and inflammation via downstream Smad-dependent mechanisms involving Smad3, Smad4, Smad7, and particularly Smad3-dependent non-coding RNAs. Targeting downstream TGF-β/Smad signaling by rebalancing Smad3/Smad7 or by specifically inhibiting or overexpressing Smad3-dependent non-coding RNAs related to fibrosis or inflammation may be a better therapeutic approach. Further studies to understand the diverse role of TGF-β signaling in kidney diseases may promote the translation from bench into clinical settings.

## Author Contributions

Y-YG, X-SL, and X-RH wrote and revised the manuscript. X-QY and H-YL revised and edited the manuscript. All authors contributed to the manuscript conception development, data collection and analysis, and discussion on the manuscript writing and revising.

## Conflict of Interest

The authors declare that the research was conducted in the absence of any commercial or financial relationships that could be construed as a potential conflict of interest.
